# Effect of different incisions on dry eye symptoms after cataract surgery in diabetic patients

**DOI:** 10.1186/s12886-025-03901-7

**Published:** 2025-02-13

**Authors:** Xiao Yang, Lijuan Li, Huiping Shen, Xue Bai

**Affiliations:** Department of Ophthalmology, The People’s Hospital of Yingshang, No. 566, Ganluo Road, Fuyang, 236200 Anhui Province China

**Keywords:** Cataract surgery; dry eye, Type 2 diabetes mellitus, Tear film stability, Anxiety, Depression

## Abstract

**Aim:**

Diabetic patients suffer from severe dry eye after cataract surgery, and the aim of this study was to investigate the effect of 2.2 mm and 3.0 mm clear corneal incisions on dry eye after phacoemulsification in patients with type 2 diabetes mellitus (T2DM).

**Methods:**

Clinical data of 104 patients with T2DM who underwent phacoemulsification in the Department of Ophthalmology of The People’s Hospital of Yingshang from January 2022 to November 2023 were retrospectively collected. The patients were categorized into 2.2 mm and 3.0 mm groups according to the surgical incision, and their preoperative and postoperative Ocular surface disease index (OSDI), Schirmer I test (SIt), and Fluorescein breakup time (FBUT), as well as anxiety and depression levels, were analyzed.

**Results:**

Before surgery, mild anxiety and depression were present in both groups. At 7 days and 1 month postoperatively, OSDI was significantly higher and SIt and FBUT were substantially lower in both groups, but the above indexes were milder in the 2.2 mm group. Meanwhile, the psychological status of both groups significantly improved at 1 month after surgery, and the improvement was more significant in the 2.2 mm group. At 3 months postoperatively, the dry eye of the two groups was relieved, and the psychological status was also improved.

**Conclusion:**

Compared with 3.0 mm, a 2.2 mm clear corneal incision effectively reduced dry eye after phacoemulsification in T2DM patients.

## Introduction

Cataract is a blinding disease that is common in the elderly population and is characterized by visual impairment due to clouding of the lens [1]. With a growing elderly population, the prevalence of cataract is increasing over the years, and as of 2020, the number of adult cataract patients in China has reached 130 million, and it is expected that by 2050, it will exceed 240 million [2, 3].

In 2021, from IDF Diabetes Atlas data, there are more than 500 million people with diabetes in the world, and more than 90% of them are type 2 diabetes mellitus (T2DM) [4, 5]. It is well known that retinopathy is the most common complication of T2DM, but in recent years, the high incidence of cataracts in T2DM patients has gained more and more attention [6]. Previous studies have shown that diabetic cataract is one of the major causes of vision loss in patients with T2DM, and the incidence of cataract in diabetic patients is about 3–5 times higher than that in non-diabetic patients, and the disease progresses more rapidly [7]. Prevention and treatment of diabetic patients with complications of cataract is of great importance.

Currently, the most common treatment for cataracts is phacoemulsification, which can significantly improve patients’ vision, but like other traditional cataract surgeries, it is prone to postoperative complications such as corneal damage and dry eye disease (DED) [8]. DED, also known as corneal conjunctival dryness, can cause to symptoms such as dryness and redness of the eyes, which can seriously affect patients’ vision recovery. It has been reported that more than one-third of patients are afflicted with DED subsequent to cataract surgery. Additionally, in comparison with non-diabetic patients, patients suffering from T2DM are more inclined to develop tear film dysfunction and more severe DED after cataract surgery [9, 10]. Research findings have indicated that the incidence of DED after phacoemulsification is influenced by the size, type, and location of the surgical incision [8, 11, 12]. Previous studies have shown that patients after phacoemulsification are less likely to develop DED and have less astigmatism in patients with smaller incisions compared to conventional cataract surgery, such as Manual small incision cataract surgery (MSICS) [13, 14, 15]. In addition, clear corneal incisions of 2.2 mm have a lower incidence of infection compared to 2.8 mm in phacoemulsification [16]. However, it is worth noting that there are various incision sizes for phacoemulsification, but fewer studies have been conducted to account for the differences in the incidence of DED in patients after phacoemulsification with different incision sizes [17].

In this study, we retrospectively analyzed the difference in the incidence of DED after phacoemulsification in T2DM patients with two different-sized incisions (2.2 mm clear corneal incisions and 3.0 mm clear corneal incisions). We statistically analyzed the baseline characteristics of the patients and their Ocular surface disease index (OSDI), Dry eye symptom score, Schirmer I test (SIt), and Fluorescein breakup time (FBUT) data at preoperative, 7 days, 1 month, and 3 months postoperatively. In addition, given that impaired vision in older adults with cataract is strongly associated with increased depression and anxiety levels, we also assessed patients’ anxiety and depression levels at various time points in this study [18]. This retrospective study aims to provide a new reference and solution idea for clinical improvement of postoperative DED after cataract surgery in patients with T2DM.

## Subjects and methods

### Clinical data

In this study, we retrospectively analyzed the clinical data of 104 eyes of 104 patients who underwent phacoemulsification for cataract treatment in the Department of Ophthalmology of The People’s Hospital of Yingshang, from January 2022 to November 2023 (If the patient had surgery in both eyes, only the first eye was analyzed). Inclusion criteria: (1) diagnosed with T2DM; (2) no medications affecting tear secretion and tear film stability within 3 months; (3) no other diseases affecting tear secretion (rheumatoid arthritis, desiccation syndrome, thyroid disorders, etc.); (4) good adherence (5) absence of severe cognitive impairment; (6) intact clinical data. Following the recommendations of the Chinese guideline for cataract surgery in adults, these patients underwent a close and comprehensive follow-up examination preoperatively (Baseline), and at 7 days, 1 month, and 3 months postoperatively [19]. We categorized the above patients into a 2.2 mm clear corneal incisions group (2.2 mm) and a 3.0 mm clear corneal incisions group (3.0 mm) based on the type of incision. There were 49 patients in the 2.2 mm group and 55 patients in the 3.0 mm group.

### Treatment program

All patients were treated with Levofloxacin Eye Drops qid for 3 days before admission. Preoperatively, the patient’s pupils were dilated using Compound Tropicamide Eye Drops, and surface anesthesia was administered intraoperatively using Proparacaine Hydrochloride Eye Drops. All procedures were performed using the same model of ultrasonic emulsifier and balanced salt solution. Prior to March 2023, cataract surgery was performed in our department using a 3.0 mm clear corneal incision, which was standard practice at the time. Since March 2023, our department has been using the 2.2 mm clear corneal incision technique because of its advantages in reducing corneal damage and improving postoperative recovery. All surgeries were performed by the same experienced surgeon, and other medical staff involved in the surgeries were from the same team, with no intraoperative complications. Postoperatively, Tobramycin and Dexamethasone Eye Ointment were administered to the affected eyes. After discharge from the hospital, Tobramycin and Dexamethasone Eye Drops and Levofloxacin Eye Drops were administered qid for 2 weeks, followed by tid for 1 week.

### Observation indicators

In the present study, we evaluated cataract grading and surgical duration in both groups. In addition, we evaluated OSDI, dry eye symptom score, SIt, FBUT, and anxiety and depression in both groups preoperatively and at 7 days, 1 month, and 3 months postoperatively.

The lens opacities classification system III (LOCS III) is a commonly used clinical criterion for cataract classification. Nuclear color and nuclear opalescence are the two most commonly used standards in LOCS III [20, 21].

The OSDI consists of 12 questions that record the patient’s subjective ocular symptoms over the past week and is used clinically to screen and diagnose DED patients and to assess the severity of their dry eye [22].

A dry eye symptom score is a rating of the severity of a patient’s subjective dry eye symptoms. 0 is asymptomatic, 0.5 is occasional, 1 is frequent and 2 is persistent. These symptoms include dry eyes, eye redness, burning sensation, foreign body sensation and eyelash adhesion discharge [23].

SIt is a commonly used method for assessing tear secretion. SIt is tested as follows: the head end of a Schirmer test strip is folded inward and placed in the conjunctival sac at the junction of the outer middle third of the lower eyelid, and the length of the test strip that is soaked with tear fluid within 5 min is measured [24].

FBUT is currently the most used method in clinical practice, and it must be conducted in an indoor environment with normal temperature, appropriate humidity, and away from direct sunlight. The standard examination procedure involves using a sterile dropper to instill 2 µL of 1% fluorescein sodium solution into the conjunctival sac, or using a fluorescein strip moistened with antibiotic eyedrops (ensuring no excess liquid remains on the strip to touch the lower eyelid margin). The patient is instructed to blink 3 to 4 times to evenly distribute the fluorescein on the ocular surface. With both eyes looking straight ahead, the time from the last blink to the appearance of the first dark spot on the cornea is recorded as the tear film breakup time. This measurement is repeated three times, and the average value is calculated [25].

The Zung Self-Rating Anxiety Scale (SAS) serves as a tool for evaluating anxiety. It comprises 20 items, with each being scored on a four-point scale spanning from “none or occasionally present” to “mostly or always present”. This self-completed (or through question-asking) questionnaire can be utilized to gauge the intensity of anxiety symptoms [26].

The Zung Self-Rating Depression Scale (SDS) is an instrument used to assess the severity of an individual’s depressive symptoms. Like the SAS, the SDS consists of 20 items, each of which is rated on a scale ranging from 1 to 4, with higher total scores indicating greater severity of depressive symptoms [27].

### Statistical analysis

We used SPSS 26 to analyze the clinical data of the two groups of patients. We first tested the normality of the measurement data, if that fulfilled the normal distribution, they were presented as x̄ ± *s*. For comparison between the two groups, the independent sample *t*-test was utilized, and the paired sample *t*-test was used for comparison between different time points within the group and preoperative period. If measures did not fulfill normal distribution, they were presented as median, quartiles [M (P25, P75)]. For comparisons between independent samples in such situations, the Mann-Whitney *U* test was adopted, and for paired samples, the Wilcoxon matched-pairs signed rank test was used. Counting data were described as numbers and percentages and were analyzed using the *χ*^2^ test. *P* < 0.05 indicates a statistically significant difference.

## Results

### Baseline characteristics were similar between the two groups

We divided 104 patients into 2.2 mm and 3.0 mm groups by distinguishing between different surgical incision sizes and subsequently statistically analyzed their demographic characteristics. As shown in Table 1, we found that there was no difference between the two groups in age, gender, education level, BMI, duration of diabetes and prevalence of other underlying diseases (*P* > 0.05). This indicates that the two groups of patients have similar baseline data, which lays the foundation for further subsequent statistical analysis.

**Table 1:** Baseline characteristics of two groups

Characteristic	2.2 mm group	3.0 mm group	χ^2^/t/Z	*P* value
**Gender [** ***n*** **(%)]**			0.100	0.752
Female	30 (61.2)	32 (58.2)		
Male	19 (38.8)	23 (41.8)		
**Age (years**, x̄**±*s*)**	72.78 ± 7.880	71.85 ± 7.347	0.617	0.539
**Educational level [** ***n*** **(%)]**			7.324	0.120
Uneducated	26 (53.1)	38 (69.1)		
Primary school	7 (14.3)	10 (18.2)		
Middle school	10 (20.4)	5 (9.1)		
High school	3 (6.1)	2 (3.6)		
University	3 (6.1)	0 (0.0)		
**BMI (**x̄**±*s*)**	24.43 ± 3.233	24.41 ± 2.739	0.034	0.973
**Diabetes duration [years**,** M (P25**,** P75)]**	10 (5, 10)	5 (3, 10)	-0.121	0.904
**Underlying disease [** ***n*** **(%)]**			0.812	0.666
Hypertension	29 (59.2)	31 (56.4)		
Hyperlipidemia	9 (18.4)	10 (18.2)		
Coronary artery disease	8 (16.3)	5 (9.1)		

### Cataract grading and surgical duration were similar between the two groups

Similarly, we analyzed the cataract grading and surgical duration of the above two groups of patients. As shown in Table 2, our results indicated that there was no significant difference in nuclear color, nuclear opalescence, and surgical duration between the two groups of patients (*P* > 0.05). Our results in this section exclude the potential impact of differences in the extent of patients’ disease and differences in the duration of surgery on subsequent statistical analyses.

**Table 2:** Cataract grading and surgical duration of two groups

Characteristic	2.2 mm group	3.0 mm group	*P* value
**Nuclear color [** ***n*** **(%)]**			0.645
≤ 3	24 (49.0)	26 (47.3)	
> 3, ≤ 5	23 (46.9)	24 (43.6)	
≥ 5	2 (4.1)	5 (9.1)	
**Nuclear opalescence [** ***n*** **(%)]**			0.560
≤ 3	22 (44.9)	27 (49.1)	
> 3, ≤ 5	24 (49.0)	22 (40.0)	
≥ 5	3(6.1)	6 (10.9)	
**Surgical duration [min** , **M (P25**,** P75)]**	14 (12, 15)	14 (12, 17)	0.422

### Patients in the 2.2 mm group had milder dry eye symptoms

After determining that the data were comparable between the two groups, we scored the dry eye symptoms and frequency at each time point in both groups. As shown in Table 3, we found that dry eye symptoms were mild in both groups preoperatively (most patients scored 0 or 0.5) and there was no significant difference between the two groups (*P* = 0.517). In contrast, on postoperative day 7, an increase in the percentage of 1–2 scores was observed in both groups, and the increase was more significant in patients in the 3.0 mm group (*P* = 0.014). At 1 month postoperatively, patients in the 3.0 mm group still had a significantly higher dry eye symptom score than those in the 2.2 mm incision group (*P* = 0.045). At 3 months postoperatively, the percentage of patients with 0 and 0.5 scores increased significantly in both groups, and the difference between the two groups was no longer significant (*P* = 0.463).

**Table 3:** Dry eye symptom score of two groups

Dry eye symptom score [*n* (%)]	2.2 mm group	3.0 mm group	*P* value
**Baseline**			0.517
0	19 (38.8)	19 (34.5)	
0.5	25 (51.0)	24 (43.6)	
1	4 (8.2)	10 (18.2)	
2	1 (2)	2 (3.6)	
**7 days**			0.014
0	4 (8.2)	4 (7.3)	
0.5	25 (51.0)	12 (21.8)	
1	15 (30.6)	30 (54.5)	
2	5 (10.2)	9 (16.4)	
**1 months**			0.045
0	10 (20.4)	6 (10.9)	
0.5	23 (46.9)	16 (29.1)	
1	13 (26.5)	25 (45.5)	
2	3 (6.1)	8 (14.5)	
**3 months**			0.463
0	12 (24.5)	15 (27.3)	
0.5	28 (57.1)	34 (61.8)	
1	9 (18.4)	5 (9.1)	
2	0 (0.0)	1 (1.8)	

While the OSDI and the dry eye symptom score are both subjective scores, the OSDI covers a broader range. Here, we also analyzed the OSDI of the two groups of patients. As shown in Table 4, we found that preoperatively, there was no significant difference in OSDI between the two groups of patients (9.35 ± 3.250 vs. 8.95 ± 2.850, *P* = 0.505). At postoperative day 7 and month 1, patients in the 2.2 incision group had significantly lower OSDI compared to the 3.0 mm incision group (26.55 ± 4.916 vs. 31.55 ± 5.750, 22.50 ± 5.499 vs. 25.05 ± 4.149, *P* < 0.001, *P* = 0.008). However, at month 3, there was no longer a significant difference in OSDI between the two groups (10.47 ± 3.802 vs. 10.10 ± 3.987, *P* = 0.635). Meanwhile, we found that OSDI was significantly higher in both groups compared to their preoperative period at postoperative day 7 and month 1 (*P* < 0.001), but recovered at postoperative month 3 (*P* = 0.130, *P* = 0.081).

**Table 4:** OSDI score of two groups

OSDI score (x̄ ± s)	2.2 mm group	3.0 mm group	*P* value
Baseline	9.35 ± 3.250	8.95 ± 2.850	0.505
7 days	26.55 ± 4.916	31.55 ± 5.750	< 0.001
1 months	22.50 ± 5.499	25.05 ± 4.149	0.008
3 months	10.47 ± 3.802	10.10 ± 3.987	0.635
*P* value	< 0.001^1^ <0.001^2^ 0.130^3^	< 0.001^1^ <0.001^2^ 0.081^3^	

^1^: Comparison to baseline at 7 days

^2^: Comparison to baseline at 1 months

^3^: Comparison to baseline at 3 months

Our data in this section show that phacoemulsification causes significant dry eye symptoms in patients, which is consistent with previous findings, but compared to 3.0 mm, clear corneal incisions of 2.2 mm resulted in more mild dry eye symptoms.

### Patients in the 2.2 mm incision group had more tear secretion

Given that decreased tear production is an important cause of DED. In this study, we evaluated tear production in both groups of patients using the SIt. As shown in Table 5, we found that both groups of patients had similar tear production preoperatively (11.75 ± 2.096 vs. 12.45 ± 2.850, *P* = 0.165). At postoperative day 7 and month 1, tear secretion was significantly greater in patients in the 2.2 mm incision group compared with patients in the 3.0 mm incision group (7.54 ± 2.449 vs. 6.38 ± 2.143, 8.25 ± 3.008 vs. 7.15 ± 2.349, *P* = 0.011, *P* = 0.039). However, at month 3, there was no longer a significant difference in tear secretion between the two groups (10.99 ± 3.280 vs. 11.40 ± 3.685, *P* = 0.547). At the same time, we found that SIt was significantly lower in both groups at postoperative day 7 and month 1 compared to baseline (*P* < 0.001), but returned to preoperative levels at month 3 (*P* = 0.191, *P* = 0.093).

**Table 5:** Schirmer test of two groups

Schirmer test (mm, x̄ ± s)	2.2 mm group	3.0 mm group	*P* value
Baseline	11.75 ± 2.096	12.45 ± 2.850	0.165
7 days	7.54 ± 2.449	6.38 ± 2.143	0.011
1 months	8.25 ± 3.008	7.15 ± 2.349	0.039
3 months	10.99 ± 3.280	11.40 ± 3.685	0.547
*P* value	< 0.001^1^ <0.001^2^ 0.191^3^	< 0.001^1^ <0.001^2^ 0.093^3^	

^1^: Comparison to baseline at 7 days

^2^: Comparison to baseline at 1 months

^3^: Comparison to baseline at 3 months

Our results showed that patients had significantly more tear secretion after a 2.2 mm incision compared to a conventional 3.0 mm incision.

### Patients in the 2.2 mm incision group had better tear film stability

Imbalance in tear film stability is also an important cause of dry eye symptoms in patients, and the main manifestation of tear film stability imbalance is shortened tear film breakup time. In this study, we evaluated the tear film breakup time in both groups of patients using FBUT. As shown in Table 6, we found that the time to tear film breakup was similar between the two groups of patients preoperatively (10.45 ± 1.549 vs. 9.84 ± 2.090, *P* = 0.098). At postoperative day 7 and month 1, patients in the 2.2 mm incision group had significantly longer tear film breakup time compared to patients in the 3.0 mm group. (5.25 ± 1.199 vs. 4.44 ± 0.850, 7.25 ± 1.749 vs. 6.34 ± 1.250, *P* < 0.001, *P* = 0.003). However, at postoperative month 3, there was no longer a significant difference in FBUT between the two groups (10.82 ± 1.450 vs. 10.34 ± 2.097, *P* = 0.179). As mentioned earlier, we also compared the data of the two groups at each time point with their data at baseline. We found that at postoperative day 7 and month 1, tear film breakup time was significantly shorter in both groups compared to their baseline (*P* < 0.001), but had returned to baseline levels by month 3 (*P* = 0.230, *P* = 0.269).

**Table 6:** FBUT of two groups

FBUT (s, x̄ ± s)	2.2 mm group	3.0 mm group	*P* value
Baseline	10.45 ± 1.549	9.84 ± 2.090	0.098
7 days	5.25 ± 1.199	4.44 ± 0.850	< 0.001
1 months	7.25 ± 1.749	6.34 ± 1.250	0.003
3 months	10.82 ± 1.450	10.34 ± 2.097	0.179
*P* value	< 0.001^1^ <0.001^2^ 0.230^3^	< 0.001^1^ <0.001^2^ 0.269^3^	

^1^: Comparison to baseline at 7 days

^2^: Comparison to baseline at 1 months

^3^: Comparison to baseline at 3 months

In the results of this section, we found that patients in the 3.0 mm group had a shorter time to tear film breakup compared to patients in the 2.2 mm incision group.

### Patients in the 2.2 mm incision group had better psychological status

Vision impairment seriously interferes with the normal life of patients and negatively affects their psychological state. In this study as shown in Table 7, we assessed the levels of anxiety and depression at various time points in both groups of patients.

**Table 7:** Psychological condition of two groups

Characteristic	2.2 mm group	3.0 mm group	*P* value
SAS (x̄ ± s)			
Baseline	57.02 ± 4.380	58.34 ± 5.250	0.168
7 days	55.73 ± 4.649	56.43 ± 4.713	0.447
1 months	51.34 ± 3.854	53.83 ± 4.875	0.005
3 months	49.75 ± 4.385	50.63 ± 4.142	0.295
*P* value	0.161^1^ <0.001^2^ <0.001^3^	0.088^1^ <0.001^2^ <0.001^3^	
**SDS [M (P25**,** P75)]**			
Baseline	56 (53, 60)	58 (53, 63.5)	0.123
7 days	58 (53, 63)	60 (54, 65.5)	0.502
1 months	50 (44, 52)	55 (47.5, 60)	0.015
3 months	49 (45, 53)	52 (45, 56.5)	0.059
*P* value	0.056^1^ <0.001^2^ <0.001^3^	0.446^1^ 0.001^2^ <0.001^3^	

^1^: Comparison to baseline at 7 days

^2^: Comparison to baseline at 1 months

^3^: Comparison to baseline at 3 months

Using the SAS scale, we found that patients in both groups were mildly depressed at baseline and postoperative day 7 and there was no significant difference (57.02 ± 4.380 vs. 58.34 ± 5.250, 55.73 ± 4.649 vs. 56.43 ± 4.713, *P* = 0.168, *P* = 0.447). At postoperative month 1, anxiety improved in both groups, but patients in the 2.2 mm group had significantly lower SAS scores (51.34 ± 3.854 vs. 53.83 ± 4.875, *P* = 0.005), although this difference was no longer significant at month 3 (9.75 ± 4.385 vs. 50.63 ± 4.142, *P* = 0.295). In addition, by comparing the data of the two groups of patients at each time point with their data at baseline, we found that there was no significant difference between the anxiety levels of the two groups and their anxiety levels at baseline at postoperative day 7 (*P* = 0.161, *P* = 0.088), whereas, at postoperative month 1 and month 3, significant improvement in the anxiety levels of the patients in both groups was observed (*P* < 0.001).

Patients’ depression levels also showed similar results to anxiety levels. Using the SDS scale, we found that patients in both groups were mildly depressive preoperatively and at postoperative day 7 and there was no significant difference (56 (53, 60) vs. 58 (53, 63.5), 58 (53, 63) vs. 60 (54, 65.5), *P* = 0.123, *P* = 0.502). At postoperative month 1, depression scores improved in both groups, and SDS scores were significantly lower in the 2.2 mm group (50 (44, 52) vs. 55 (47.5, 60), *P* = 0.015), although this statistically significant difference was no longer evident after 3 months (49 (45, 53) vs. 52 (45, 56.5), *P* = 0.059). In addition, by comparing the data of the two groups at each time point with their data at baseline, we found that there was no significant difference between the depression levels of the two groups and their depression levels at baseline at postoperative day 7 (*P* = 0.056, *P* = 0.446), whereas a significant improvement in the depression levels of both groups was observed at postoperative month 1 and month 3 (*P* < 0.001).

Our results suggest that phacoemulsification significantly enhances the psychological status of patients with T2DM cataracts and that a 2.2 mm incision has a more positive impact on the psychological status of patients compared to a 3.0 mm incision.

## Discussion

Previous studies showed that the occurrence rate of dry eye following cataract surgery stood at 37.4%, and these patients may cause irreversible damage if not treated in time [9, 28]. Notably, T2DM patients are more likely to develop complications including DED after cataract surgery compared to non-diabetic patients [10, 29, 30]. In this study, we found that a 2.2 mm incision was more effective in improving patients’ dry eye symptoms compared to a 3.0 mm clear corneal incision. These improvements were primarily reflected in lower dry eye symptom scores and OSDI scores as well as higher SIt and FBUT results in patients of the 2.2 mm incision. Additionally, we noted that both incision sizes led to significant improvements in the patients’ adverse psychological status. However, the patients in the 2.2 mm group exhibited faster rates of recovery in this regard.

Currently, commonly used cataract extraction surgeries include extracapsular cataract extraction (ECCE), MSICS, and phacoemulsification. ECCE and MSICS have the strongpoint of relatively simpler operation, lower cost, and faster recovery, but they also have disadvantages, including a larger incision, slower recovery of postoperative visual acuity, and more pronounced postoperative astigmatism [31]. As cataract surgical techniques continue to evolve, these two traditional surgical modalities have been gradually replaced by phacoemulsification with fewer complications in more developed regions [15, 31].

The incidence of DED in patients after phacoemulsification has been reported to be about half that of MSICS with better visual recovery [13, 32]. Recent studies have found that reducing the size of the phacoemulsification incision reduces postoperative astigmatism and inflammation, as well as decreasing operative time and postoperative recovery time [16, 33]. In addition, a recent study has shown that longer surgical incisions are an independent risk factor for the development of dry eye in patients following cataract phacoemulsification [34]. In the present study, we found that dry eye symptoms were milder after phacoemulsification with 2.2 mm clear corneal incisions in patients with T2DM compared to 3.0 mm clear corneal incisions, although this difference was no longer significant after 3 months postoperatively, while the anxiety and depression levels of patients in the group with a 2.2 mm incision showed remarkable improvement in comparison to those in the 3.0 mm group during this period. Our findings suggest that it is feasible to prevent postoperative DED in patients with T2DM by reducing the incision size for phacoemulsification.

Cataract surgery can effectively improve patients’ vision, but it can cause damage to the corneal conjunctiva, leading to tear film instability, and many cataract patients develop DED after surgery [8]. Previous studies have shown that before and after phacoemulsification surgery, diabetic patients have poorer tear film stability compared to non-diabetic patients [10]. Compared to MSICS, patients after phacoemulsification have better tear film stability [35]. Meanwhile, another study also showed that patients had longer FBUT from 1 to 12 weeks after phacoemulsification compared to conventional MSICS [36]. During this research, we discovered that patients in the 2.2 mm incision group had longer tear film breakup time after phacoemulsification compared with the 3.0 mm incision. Our results suggest that decreasing the size of clear corneal incisions from 3.0 mm to 2.2 mm may be effective in improving the ocular environment after phacoemulsification.

Another important cause of DED is inadequate tear production [19]. Previous studies have shown that cataract patients produce more tears after phacoemulsification compared to MISUS, and this difference persists up to 3 months postoperatively [36]. Consistent with the above study, we found in the present study that patients in the 2.2 mm incision group had more tear production postoperatively compared to the 3.0 mm incision group, and this difference persisted for up to one month. We speculate that smaller incisions may have less impact on corneal nerves, and local inflammation and therefore less impact on tear secretion and tear film stability, which may explain these results [15, 17].

Many studies have pointed out that diminished vision or even blindness due to cataracts is significantly linked to a heightened probability of suffering from anxiety and depression in the elderly [18, 37, 38]. We found in the present study that preoperatively, patients in both groups were in a mild state of anxiety and depression (SAS and SDS scores greater than 50), which could be attributed to the patient’s fear of their vision problems. One month after phacoemulsification, both groups showed significant improvement in anxiety and depression scores, and the improvement was more pronounced in the 2.2 mm group. We believe that the improvement in anxiety and depression levels in both groups was mainly due to the improvement in vision from cataract surgery. Recent reports suggest that DED can lead to significant adverse psychological conditions in patients [39, 40]. In addition, refractive surgery patients experience significant dry eye symptoms, anxiety, and depression postoperatively, and that such poor psychological conditions may be related to the postoperative dry eye; whereas DED after refractive surgery has been attributed primarily to corneal damage from the surgery [41]. Therefore, we hypothesized that the reason for the favorable psychological profile of patients in the 2.2 mm group over the 3.0 mm group was related to milder dry eye symptoms due to the smaller incision.

In summary, we found in this retrospective study that compared to phacoemulsification with a 3.0 mm clear corneal incision, a 2.2 mm incision was effective in improving patients’ dry eye symptoms and patients’ adverse psychological status after surgery. In conclusion, decreasing the incision size has a positive impact on improving dry eye symptoms in cataract patients after surgery. Therefore, the use of smaller incision sizes should be emphasized in clinical practice.

Although the results of this study are encouraging, some limitations remain. First, we were unable to fully assess the potential impact of topical eye drop use on dry eye symptoms. In the future, more rigorous research should be designed to clarify the specific effects of eye drops on dry eye symptoms. Second, patients’ behavioral status may have been a confounding factor during this study. Although we considered the baseline characteristics of the patients in our statistical analysis, there may have been other factors not included that influenced dry eye symptoms. Future studies should use more comprehensive behavioral assessment tools and control for these influences as much as possible. In addition, the sample size of this study was relatively small, and further studies in larger patient populations are warranted.

## Data Availability

Data are available from the corresponding author under sound reasoning.
